# Design of a novel curcumin-soybean phosphatidylcholine complex-based targeted drug delivery systems

**DOI:** 10.1080/10717544.2017.1303855

**Published:** 2017-04-24

**Authors:** Jiajiang Xie, Yanxiu Li, Liang Song, Zhou Pan, Shefang Ye, Zhenqing Hou

**Affiliations:** 1 Xiamen Xianyue Hospital, Xiamen, China,; 2 Department of Biomaterials, College of Materials, Xiamen University, Xiamen, China, and; 3 Department of Physics, Changji University, Changji, China

**Keywords:** Curcumin, anticancer drug-phospholipid complex, nanoparticles, self-assembly, targeting

## Abstract

Recently, the global trend in the field of nanomedicine has been toward the design of combination of nature active constituents and phospholipid (PC) to form a therapeutic drug-phospholipid complex. As a particular amphiphilic molecular complex, it can be a unique bridge of traditional dosage-form and novel drug delivery system. In thisarticle, on the basis of drug-phospholipid complex technique and self-assembly technique, we chose a pharmacologically safe and low toxic drug curcumin (CUR) to increase drug-loading ability, achieve controlled/sustained drug release and improve anticancer activity. A novel CUR-soybean phosphatidylcholine (SPC) complex and CUR-SPC complex self-assembled nanoparticles (CUR-SPC NPs) were prepared by a co-solvent method and a nanoprecipitation method. DSPE-PEG-FA was further functionalized on the surface of PEG-CUR-SPC NPs (designed as FA-PEG-CUR-SPC NPs) to specifically increase cellular uptake and targetability. The FA-PEG-CUR-SPC NPs showed a spherical shape, a mean diameter of about 180 nm, an excellent physiological stability and pH-triggered drug release. The drug entrapment efficiency and drug-loading content was up to 92.5 and 16.3%, respectively. *In vitro* cellular uptake and cytotoxicity studies demonstrated that FA-PEG-CUR-SPC NPs and CUR-SPC NPs presented significantly stronger cellular uptake efficacy and anticancer activity against HeLa cells and Caco-2 cells compared to free CUR, CUR-SPC NPs and PEG-CUR-SPC NPs. More importantly, FA-PEG-CUR-SPC NPs showed the prolonged systemic circulation lifetime and enhanced tumor accumulation compared with free CUR and PEG-CUR-SPC NPs. These results suggest that the FA targeted PEGylated CUR-SPC complex self-assembled NPs might be a promising candidate in cancer therapy.

## Introduction

Cancer continues to be a prevalent and lethal disease worldwide. Complete surgical resection would still be one of the best options to treat cancer for a long-term survival, with a 5-year survival rate of 20–40% (Siegel et al., 2015). Unfortunately, only a small percentage of patients suffering from cancer are suitable for surgery at the proper time, even so, they are also encountered with a high rate of tumor recurrence (Rizvi & Gores, 2013). In addition, radiotherapy and chemotherapy would still be widely used to treat the residual, infiltrative tumor cells after surgical resection (Rose et al., 1999; Chou, 2006). But the chemotherapeutic efficacy is largely limited by the nonspecific cellular uptake and tissue biodistribution, rapid metabolization and excretion from the body, serious side effects, poor tumor selectivity and low-tumor cell uptake efficiency of anticancer drugs (Chabner & Roberts, 2005; Cheng et al., 2015; Li et al., 2016). Therefore, the development of novel cancer chemotherapy and drug delivery is urgently needed.

In recent decades, curcumin (CUR), [1,7-bis(4-hydroxy-3-methoxyphenyl)-1,6-heptadiene-3,5-dione], a yellow natural polyphenol extracted from turmeric (Curcuma longa), has attracted considerable research interest because of its various health benefits ranging from potent antitumor/anti-inflammatory/antioxidant properties (Balogun et al., 2003; Duvoix et al., 2005; Aggarwal et al., 2007; Reuter et al., 2008) and hepatoprotective pharmacological properties (Aggarwal et al., 2003). The anticancer potential of CUR derived from its ability to suppress proliferation of a wide variety of tumor cells including breast carcinoma, cervical carcinoma, myeloid leukemia, hepatocellular carcinoma, melanoma, etc (Kuo et al., 1996; Anirudhan & Binusreejayan, 2016). Furthermore, CUR has been certified as a safe active compound by preclinical and clinical studies even at a very high dose (Anitha et al., 2012). Despite number of activities and high-safety profile of CUR, the limitations, such as poor aqueous solubility and low-systemic bioavailability are still some obstacles that need to be overcome, which interns limit its therapeutic efficacy (Dhule et al., 2012). Furthermore, for anticancer treatment, the drug molecule should present at the site of tumor for longer period of time to exert its therapeutic effect (Byrne et al., 2008). Thus, the improvement of solubility and the release/bioavailability become an urgent and necessary issue.

To addresses this problem, various strategies have been developed, including salt formulations, crystal engineering (Blagden et al., 2007), solid dispersions (Zhang et al., 2014), prodrug strategies (Stella & Nti-Addae, 2007), cyclodextrin complexation, and phospholipid complexation (Khan et al., 2013; Li et al., 2015b; Ahmad et al., 2016) and so on. Among these strategies, phospholipid complexation becomes one of the most prevailing strategies to improve the solubility of drug, increase drug-loading capacity and control the release rate of drug (Ma et al., 2013; Khan et al., 2013; Ahmad et al., 2016; Khatik et al., 2016). It is well known that phospholipid is a vital component of cell membrane with good biocompatibility/biodegradability and low toxicity. At present, drug-phospholipid complex has received significant attention due to the superior amphiphilic characteristic of phospholipid and the convenience, flexibility and versatility of manufacturing process (Khan et al., 2013; Liu et al., 2015; Li et al., 2016). So, drug-phospholipid complex was an advantageous option to improve both the solubility and permeability of therapeutic drug. A variety of therapeutic drug-phospholipid complexes (e.g. others reported antidiabetic drug insulin-phospholipid complex (Cui et al., 2006), hepatoprotective drug CUR-phospholipid complex (Maiti et al., 2007) and our reported anticancer drug mitomycin C-phospholipid complex (Hou et al., 2013), etc.) have been previously investigated, in which the drug molecules are entrapped within the phospholipid molecules. In the previous study, we first reported chemotherapeutic drug (mitomycin C)-phospholipid complex self-assembled phytosomal NPs (Hou et al., 2013). Compared to the free drug, these phytosomal NPs improved drug stability, increased drug-loading capacity and enhanced drug delivery efficacy. In addition, a phytosome unit is similar to a liposome in general, but with a different guest localization. In a liposome, the ingredients are hosted in the inner cavity, with limited possibility of molecular interaction between the surrounding lipid and a hydrophilic guest. In a phytosome, the ingredients are dispersed into phospholipid (a dietary surfactant and can be compared to an integral part of the lipid membrane), where the polar groups of the hydrophilic guest interact with the phosphate heads of phospholipid via electrostatic and hydrogen-bonding interactions (Kidd, 2009; Li et al., 2015a,b). Therefore, phytosomal nanoparticle, a type of drug-phospholipid complex-based drug delivery system, possesses lower lipid material dosage and higher drug-loading ability compared to a liposomal nanoparticle for increasing the therapeutic drug effectiveness.

**Figure 1.**
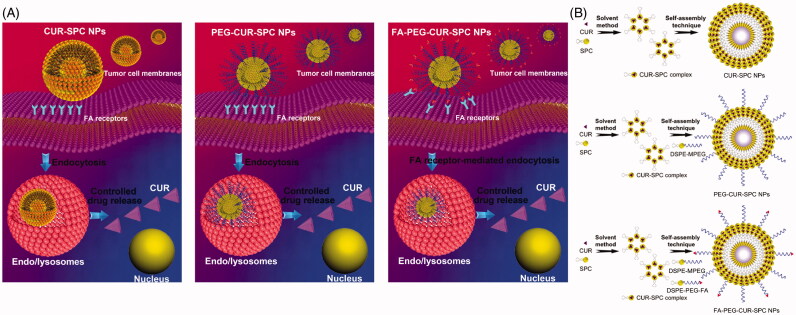
(A) Schematic illustration of cellular uptake and drug release of CUR-SPC complex-based self-assembled NPs (CUR-SPC NPs, PEG-CUR-SPC NPs and FA-PEG-CUR-SPC NPs). (B) Schematic illustration of synthesis of CUR-SPC complex by a co-solvent method and preparation of CUR-SPC complex-based self-assembled NPs (CUR-SPC NPs, PEG-CUR-SPC NPs and FA-PEG-CUR-SPC NPs) by a self-assembly technique.

In this study, we developed a CUR-phospholipid complex-based therapeutic system and further introduced DSPE-MPEG/DSPE-PEG-folate (DSPE-PEG-FA) for surface functionalization to guide the system to the site of interest and maximize its therapeutic outcomes (Figure 1). Initially, CUR was complexed with soybean phosphatidylcholine (SPC) by a co-solvent procedure to increase the solubility, improve the amphiphilicity and enhance the stability of CUR drug. Then, based on CUR-SPC complex, the FA targeted PEGylated CUR-SPC complex self-assembled NPs were constructed via a straightforward and robust nanoprecipitation method by hydrophilic-hydrophobic interaction-induced self-assembly. In this system, the outer PEG protection shell and FA targeting ligand could endow it with good stability in physiological media, extended blood circulation time and enhanced tumor accumulation/cellular uptake, while the inner CUR-SPC complex could contribute to a high-loading capability of CUR for reducing the drug release and enhancing the anticancer activity. Following this, the *in vitro and in vivo* studies including physicochemical characteristics, drug-loading ability, drug stability, drug release, cellular uptake, cytotoxic effect, subcellular distribution, pharmacokinetics, and tumor accumulation of those drug delivery systems were systemically investigated.

## Experimental

### Materials

CUR was purchased from Aladdin Industrial Corporation (Shanghai, China). 1, 2-distearoyl-sn-glycero-3-phosphoethanolamine-N-[methoxy(polyethylene glycol)-2000] (DSPE-MPEG) and 1, 2-distearoyl-sn-glycero-3-phosphoethanolamine-N-[folate(polyethylene glycol)-2000] (DSPE-PEG-FA) (ammonium salt) was purchased from Sinopeg Biotech Co., Ltd. Soybean phosphatidylcholine (SPC, LIPOID S-100) consisting of 90–95% phosphatidylcholine was obtained by Lipoid GmbH (Germany). Cy5.5 was from Molecular Probes Inc. (Eugene, OR). Toluene and tetrahydrofuran (THF) were obtained from Sinopharm Chemical Reagent Co., Ltd. (Shanghai, China). RPMI-1640, penicillin-streptomycin, and trypsin were ordered from Sigma Chemical Corp (St. Louis, MO). All other chemicals and reagents were purchased from Sigma-Aldrich (St. Louis, MO) unless otherwise noted.

### Synthesis of amphiphilic CUR-SPC complex

The CUR-SPC was synthesized by a co-solvent technique based on our previous reported study with some modifications.[31] Firstly, 10 mg of CUR and 40 mg of SPC were added into 12 mL of tetrahydrofuran (THF) and continuously stirred at 40 °C. The resulting mixture became clear and transparent in 4 h, indicating that the CUR-SPC complex dispersion was formed. Subsequently, the organic solvent was removed through vacuum rotary evaporation, obtaining the film of the crude CUR-SPC complex. The physical mixture of CUR and SPC was prepared at the same weight ratio by grinding them inside an agate mortar.

In order to determine the complexation rate (CR) of CUR and SPC in the formation of the CUR-SPC complex, the crude CUR-SPC complex was added into toluene (insoluble solvent of CUR), followed by the vigorous vortexing, and filtered through a 220 nm pore-size membrane filter to remove the excess and uncomplexed CUR. The filtrate was evaporated to dryness according to HPLC analysis. The CR of the CUR-SPC complex was calculated by the following equation:

CR(%)=W(complexed)/W(total)× 100%

W (complexed): the amount of the complexed CUR. W (total): the total amount of CUR added in the synthesis of CUR-SPC complex.

### Self-assembly of amphiphilic FA-PEG-CUR-SPC complex

The FA-PEG-CUR-SPC NPs were constructed by the amphiphilic CUR-SPC complex, DSPE-MPEG and DSPE-PEG-FA by a nanoprecipitation method and a self-assembly technique. Briefly, the CUR-SPC complex (equivalent to 10 mg of CUR), 4 mg DSPE-MPEG and 2 mg of DSPE-PEG-FA were added into 10 mL of THF, then the mixture was added to the DI water dropwise under fast stirring. The organic solvent was removed via stirring overnight followed by vacuum rotary evaporation. The suspensions were further purified via ultrafiltration. After that, the resulting FA-PEG-CUR-SPC NPs extruded through by extrusion through 220 nm pore-size membrane filters. On the other hand, the CUR-SPC NPs was prepared using the same procedures as above except that the DSPE-PEG was removed, and the PEG-CUR-SPC NPs was also prepared with the similar procedures except that DSPE-PEG-FA was replaced by DSPE-MPEG at the equivalent mole of DSPE. For *in vitro* cytotoxicity, the drug-free CUR-SPC NPs, drug-free PEG-CUR-SPC NPs and drug-free FA-PEG-CUR-SPC NPs were prepared using the same procedures as above except that the CUR-SPC complex was replaced by the free CUR.

### Physicochemical characterization of amphiphilic CUR-SPC complex

The thermograms were recorded by a differential scanning calorimetry (DSC) 204F1 (Netzsch, Selb, Germany). The X-ray diffraction (XRD) spectra were recorded by an X-ray diffractometer (Phillips X’ pert Pro Super, Panalytical, Almelo, Netherlands). The Fourier transform infrared spectrum was performed on a Bruker IFS-55 infrared spectrometer (Bruker, Zurich, Switzerland). The morphology was observed by transmission electron microscopy (TEM, JEM 1400, JEOL, Tokyo, Japan). The free CUR, SPC and physical mixture of CUR and SPC were used as controls for comparison.

### Drug-loading content

The amount of the drug was determined by HPLC analysis. The drug-loading content of the FA-PEG-CUR-SPC NPs, PEG-CUR-SPC NPs and CUR-SPC NPs was calculated by the following equation:

Drug-loading content (%) = Amount of drug in the NPs/Amount of the NPS × 100%

### Physicochemical characterization of self-assembled FA-PEG-CUR-SPC NPs

The hydrodynamic particle size and polydispersity index (PDI) was assayed by dynamic light scattering (DLS) using a Malvern Zetasizer Nano-ZS (Malvern Instruments, Worcestershire, UK). The zeta potential was determined by electrophoretic light scattering (ELS) using a same equipment. The morphology was visualized using scanning electron microscopy (SEM, LEO 1530VP, Oberkochen, Germany) and transmission electron microscopy (TEM, JEM 1400, JEOL, Tokyo, Japan). The CUR-SPC NPs and PEG-CUR-SPC NPs were used as controls.

### *In vitro* stability tests

The *in vitro* stability of the FA-PEG-CUR-SPC NPs was performed under different media by incubating the FA-PEG-CUR-SPC NPs in water and phosphate-buffered saline (PBS) solution for 48 h, respectively. The hydrodynamic particle size and residual drug-loading content of the FA-PEG-CUR-SPC NPs was determined at predesigned time intervals by DLS analysis. The CUR-SPC NPs and PEG-CUR-SPC NPs were used as controls.

### *In vitro* release profiles

The release profile of CUR drug from the FA-PEG-CUR-SPC NPs was evaluated by a dialysis technique using a dialysis membrane (molecular weight cutoff of 3500 Da). The experiment was evaluated in PBS solution (pH 7.4, 6.5 and 5.5). A total of 1 mL of the FA-PEG-CUR-SPC NPs was transferred into a dialysis membrane and then immersed into 49 mL of PBS at 37 °C with gentle shaking. At the predesigned time intervals, 1 mL of the release medium was withdrawn and subsequently the release medium was replaced with 1 mL of fresh medium. The concentration of released CUR was determined by a HPLC method as described above. The accumulative drug release of the FA-PEG-CUR-SPC NPs was expressed as a percentage of the released drug. The free CUR, CUR-SPC NPs and PEG-CUR-SPC NPs were used as controls for comparison.

### *In vitro* cellular uptake

Human cervical cancer cell line HeLa cells were cultured in 6-well plates with at a density of 1 × 10^5^ cells per well. The cells were incubated at 37 °C and 5% CO_2_ for 24 h. The FA-PEG-CUR-SPC NPs suspensions were added to the cells for predesigned incubation time periods. After incubation, the cells were washed with PBS, fixed with 4% paraformaldehyde, stained with DAPI and imaged using a Leica TCS SP5 confocal laser scanning microscopy (Leica Microsystems, Mannheim, Germany). The cells incubated with the free CUR, PEG-CUR-SPC NPs and FA-PEG-CUR-SPC NPs at the equivalent concentration of CUR were used as controls for comparison.

### Flow cytometry analysis

HeLa cells were seeded in 6-well plates with a density of 1 × 10^5^ cells/mL and incubated for 24 h. After incubation, HeLa cells were then treated with the FA-PEG-CUR-SPC NPs for 4 h. After treatment, HeLa cells were harvested with trypsin-EDTA and washed with PBS. Then the fluorescence intensity was measured using a Beckman Coulter Cell Lab Quanta SC. HeLa cells treated with the free CUR, CUR-SPC NPs and PEG-CUR-SPC NPs at the equivalent concentration of CUR were used as controls for comparison.

### *In vitro* cell cytotoxicity assay

The *in vitro* cytotoxicity of the FA-PEG-CUR-SPC NPs was measured using a 3-(4, 5-dimethylthiazol-2-yl)-2, 5-diphenyltetrazolium bromide (MTT) assay according to the manufacturer’s suggested procedures. HeLa cells or colon cancer cell line Caco-2 cells were incubated with the FA-PEG-CUR-SPC NPs at different CUR concentrations for 24 and 48 h at 37 °C and 5% CO_2_. The data was expressed as the percentage of surviving cells. The cells treated with the free CUR, CUR-SPC NPs and PEG-CUR-SPC NPs at the equivalent concentration of CUR were used as controls for comparison. In addition, the cells incubated with the drug-free CUR-SPC NPs, drug-free PEG-CUR-SPC NPs and drug-free FA-PEG-CUR-SPC NPs at different NPs concentrations were used for evaluation of *in vitro* biocompatibility of drug-free NPs.

### *In vivo* pharmacokinetics

All animal experiments were conducted under protocols approved by the Animal Care and Use Committee (CC/ACUCC) of Xiamen University. Adult male SD rat weighing 200 ± 20 g (mean ± SD) were supplied by the Experiment Animal Center of Xiamen University. The PEG-CUR-SPC NPs and FA-PEG-CUR-SPC NPs at an equivalent dose of CUR (4 mg/kg) were administered into rats (*n* = 4) by intravenous injection. Blood samples were withdrawn from the postorbital venous and collected into heparinized tubes at the predesigned time following the injection. The blood samples were centrifuged immediately at 3000 rpm for 15 min at 4 °C to harvest the plasma and stored at −20 °C until analysis by HPLC.

### *In vivo* fluorescence imaging of HeLa tumor-bearing mice

A suspension of 5 × 10^6^ of HeLa cells in PBS (80 μL) was injected subcutaneously into athymic (nu/nu) mice (7 weeks old, female, 16 − 18 g). Cy5.5 as a lipophilic and hydrophobic near-infrared fluorescent probe was encapsulated within the PEG-CUR-SPC NPs and FA-PEG-CUR-SPC NPs, respectively (designed as Cy5.5-PEG-CUR-SPC NPs and Cy5.5-FA-PEG-CUR-SPC NPs, respectively). When tumors reached a volume of 60 − 80 mm^3^, 200 μL of Cy5.5-PEG-CUR-SPC NPs and Cy5.5-FA-PEG-CUR-SPC NPs at the same concentration of Cy5.5 (0.2 mg/kg) was injected via the tail vein, respectively. Real-time whole-body fluorescence imaging was recorded with using a Maestro *in vivo* imaging system (Cambridge Research & Instrumentation, Woburn, MA). Images were obtained at different time points after intravenous injection. After 24 h post-injection, the mice were sacrificed. Then the tumor and major organs (liver, heart, spleen, kidney and lung) were collected for the *ex vivo* fluorescence imaging and analyzed using by Living Image software (Caliper Life Sciences, Hopkinton, MA).

### Statistical analysis

All experiments were repeated at least three times. All data were expressed as mean ± SD. Statistical tests were performed by the Student’s *t*-test. The statistical difference would be considered statistically significant when the *p* value was less than 0.05.

## Results and discussion

### Synthesis of CUR-SPC complex and preparation of CUR-SPC complex self-assembled FA-PEG-CUR-SPC NPs

The synthetic route of CUR-SPC complex and the preparation of CUR-SPC complex self-assembled NPs (CUR-SPC NPs, PEG-CUR-SPC NPs and FA-PEG-CUR-SPC NPs) are illustrated in Figure 1(B). CUR-SPC complex was first synthesized by a co-solvent method using CUR and SPC. Subsequently, the CUR-SPC complex self-assembled FA targeted PEGylated NPs were constructed by the CUR-SPC amphiphilic complex, DSPE-MPEG lipid-polymer conjugate, and DSPE-PEG-FA lipid-polymer-targeting ligand conjugate via a nanoprecipitation method and a self-assembly technique. It was expected that the anticancer drug-phospholipid complex self-assembled NPs could not only improve the amphiphilicity of CUR drug for enhancing drug-loading capacity and reducing serious drug release. Furthermore, PEGylation and FA functionalization could improve the physiological stability of CUR-SPC NPs, extend the blood circulation time, and specifically enhance the cellular uptake and intracellular drug delivery efficiency.

### Characterization of CUR-SPC complex

**Figure 2.**
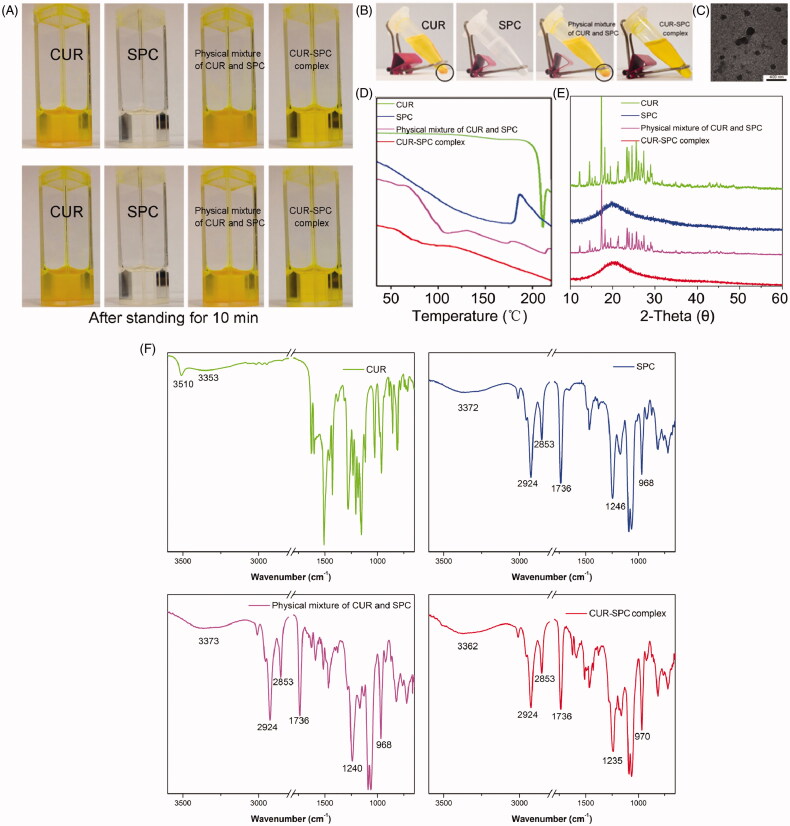
(A) The photographs of CUR, SPC, physical mixture of CUR and SPC and CUR-SPC complex dispersed in organic solvent (toluene) after preparation. (B) The photographs of CUR, SPC, physical mixture of CUR and SPC and CUR-SPC complex dispersed in organic solvent (toluene) after 2 h of storage. (C) TEM image of CUR-SPC complex. (D) DSC spectra, (E) XRD spectra and (F) FTIR spectra of CUR, SPC, physical mixture of CUR and SPC and CUR-SPC complex.

CUR is difficult to dissolve in toluene directly. Moreover, CUR could be precipitated in toluene after 10 min of storage (Figure 2A). Furthermore, the solubility of CUR in toluene could not be enhanced by simple physical mixing with SPC. Interestingly, the prepared CUR-SPC complex (CR was determined as high as 90.2 ± 2.4%) is well soluble in toluene and stable after 1 h of storage (Figure 2A and B). The result indicated that the drug-phospholipid complex technique exerted the solubilization effect of CUR. When the CUR-SPC complex was added in the non-polar solvent, the polar head of SPC was oriented inward to CUR drug, whereas the non-polar tail of SPC was oriented outward to the organic phase, obtaining the nearly spherical reversed micelles (TEM image is seen in Figure 2C). We then used CUR, SPC, physical mixture of CUR and SPC, CUR-SPC complex by differential scanning calorimetry (DSC), X-ray diffractometry (XRD), Fourier transform infrared spectroscopy (FT-IR) to investigate the distribution state of CUR drug in the pharmaceutical matrix of CUR-SPC complex.

First, the DSC spectrum of CUR showed a sharp peak (208 °C), and that of SPC showed a wide endothermic peak (150–200 °C) (Figure 2D). The DSC spectrum of the physical mixture of CUR and SPC clearly demonstrated additive effect of CUR and SPC, although with less intensity. Compared to CUR, SPC and their physical mixture, the original peaks of CUR disappeared in the CUR-SPC complex. The result implied that CUR interacted with SPC by some weak molecular interactions (such as electrostatic attraction, hydrogen bond and so on) in the CUR-SPC complex, which might change the crystalline structure of CUR. This explanation could be further confirmed in the XRD result.

Second, the XRD spectrum of CUR and SPC, respectively, exhibited some sharp crystalline peaks and a broad peak (Figure 2E), indicating a crystalline form of CUR and an amorphous one of SPC. Additionally, the XRD spectrum of the physical mixture clearly still detected some crystalline peaks, which indicated that CUR in a crystalline form still remained in the physical mixture of CUR and SPC. Nevertheless, the crystalline signals almost disappeared in the XRD spectrum of the CUR-SPC complex. This result further revealed that CUR uniformly dispersed in the SPC matrix.

Third, the FT-IR spectrum of the physical mixture of CUR and SPC showed an additive effect compared with that of CUR or SPC **(**Figure 2F). Whereas, the characteristic absorption peak (3510 cm^−1^, O–H stretching vibration) of CUR fused with a wide absorption peak (3372 cm^−1^) of SPC, shifting to a lower wavenumber at 3362 cm^−1^ of CUR-SPC to form a wider absorption peak. And, the P = O stretching vibration of SPC at 1246 cm^−1^ in the FT-IR spectrum of the SPC shifted to a lower wavenumber at 1235 cm^−1^ in that of the CUR-SPC complex. It could be further explained that the phenol hydroxyl group of CUR interacted with the phosphate group of SPC by hydrogen bonding in construct of the CUR-SPC complex. In addition, the absorption peaks of CN^+^− (CH_3_)_3_ rocking vibration of SPC at 968 cm^−1^ shifted to a higher wavenumber in the spectrum of the CUR-SPC complex. As the N atoms of SPC were good electron donors prone to being positively charged, while the O atoms of CUR were good electron acceptors prone to being negatively charged, charge-transfer reactions were easy to develop between SPC and CUR under external environment. Thus the result also implied the presence of electrostatic interactions between CUR and SPC. On the other hand, the absorption peak of non-polar group of SPC with two long fatty acid tails at 2925, 2853 and 1736 cm^−1^ corresponding to C–H and C=O stretching vibration was not associated to any change in the spectrum of the CUR-SPC complex. This result suggested that the long-chain fatty acids were not participated in the formulation of CUR-SPC complex.

Taken together, these results demonstrated the existence of multiple weak molecular interactions (hydrogen bond and electrostatic interactions) between CUR and polar parts of SPC, which led to the highly effective formulation of the CUR-SPC complex rather than just a simple physical mixture (Cui et al., 2006; Kidd, 2009). Moreover, the non-polar part of SPC not participated in their complexation could turn freely and enwrap the polar parts to be assembled into the reverse micelles. In other words, the hydrophilic head of CUR and SPC tend to the inner space, while the hydrophobic head of SPC tend to the outer space, leading to a good state of CUR highly dispersed in SPC. This might be facilitated for the improvement in amphiphilicity of CUR drug (Cui et al., 2006).

### Characterization of CUR-SPC complex self-assembled targeted NPs (FA-PEG-CUR-SPC NPs)

**Figure 3.**
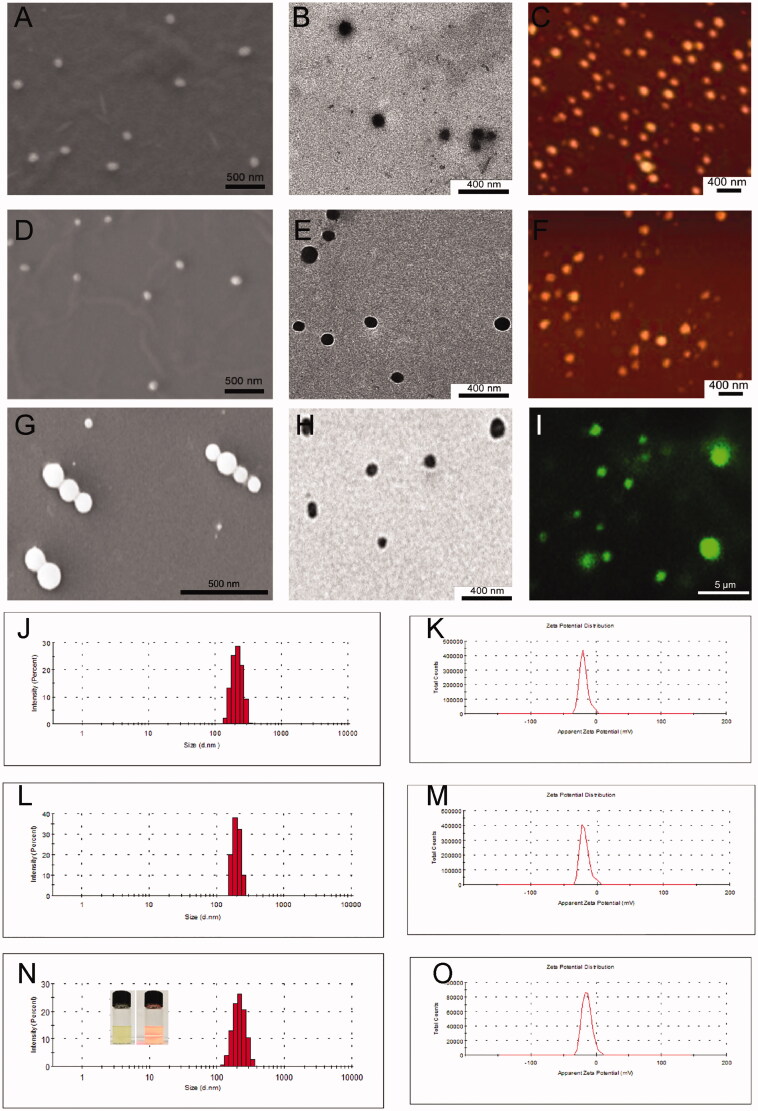
(A) SEM image, (B) TEM image and (C) AFM image of the self-assembled CUR-SPC NPs. (D) SEM image, (E) TEM image and (F) AFM image of the self-assembled PEG-CUR-SPC NPs. (G) SEM image, (H) TEM image and (I) confocal laser scanning microscopy image of the self-assembled FA-PEG-CUR-SPC NPs. (micro-sized FA-PEG-CUR-SPC NPs could be formulated under certain experimental parameters (low amplitude and high concentration of CUR-SPC complex). (J) Hydrodynamic particle size distribution and (K) zeta potential of CUR-SPC NPs. (L) Hydrodynamic particle size distribution and (M) zeta potential of PEG-CUR-SPC NPs. (N) Hydrodynamic particle size distribution and (O) zeta potential of FA-PEG-CUR-SPC NPs (Inset of N: photograph and Tyndall effect of FA-PEG-CUR-SPC NPs).

DLS, ELS, scanning electron microscopy (SEM), TEM, atomic force microscopy (AFM) and confocal laser scanning microscopy were then used to evaluate the CUR-SPC complex self-assembled NPs (Figure 3). Because of the amphiphilicity of CUR-SPC complex, the FA targeted PEGylated CUR-SPC complex can be self-assembled into micellar structures in water environment. The Tyndall effect of the micellar dispersion under a laser beam provides the first indication for the formation of FA-PEG-CUR-SPC NPs (inset of Figure 3N). SEM, TEM and confocal laser scanning microscopy images showed that the morphology of the FA-PEG-CUR-SPC NPs exhibited a spherical particle shape; a compact structure and a homogeneous particle distribution (Figure 3G, H and I). The hydrodynamic particle size of the FA-PEG-CUR-SPC NPs was 185.3 ± 4.2 nm a polydispersity (PDI) of 0.152 **(**Figure 3N**)**. The nanoscaled particle size with a narrow particle size distribution is useful for cancer therapy via enhanced penetration and retention (EPR) effect, ensuring the passive tumor targeting mechanism (Peer et al., 2007; Torchilin, 2011). The surface charge of the FA-PEG-CUR-SPC NPs was −15.7 ± 4.4 mV (Figure 3O), which is facilitated for stabilizing the NPs as well as decreasing the nonspecific interactions with negative charged red blood cells and serum proteins (Li et al., 2014). In addition, the hydrodynamic particle size and surface charge of CUR-SPC NPs and PEG-CUR-SPC NPs are shown in Table S1 in the Supporting Information. Furthermore, it is worth mentioning that the PEG-CUR-SPC NPs and FA-PEG-CUR-SPC NPs also exhibited excellent stability with no significant particle size change and residual drug-loading content in aqueous media including Milli-Q water and phosphate buffer saline (PBS) for 120 h compared to CUR-SPC NPs **(**Figure S1 in the Supporting Information). However, the CUR-SPC NPs showed a poor physiological stability, such as some precipitate, phase separation, obvious increase of hydrodynamic particle size and significant reduction of residual drug-loading content. These results implied that the introduction of lipid-PEG conjugate significantly improve of long-term stability of the PEG-CUR-SPC NPs and FA-PEG-CUR-SPC NPs.

Most of the CUR anticancer drug molecules was introduced within the SPC complex by multiple weak molecular interactions and subsequently the PEGylated CUR-SPC complex assembled into the NPs system with amorphous form, which could be expected to realize the high drug-loading efficiency, low-drug burst release and excellent cellular uptake effect (discussed below).

### Drug-loading capacity

CUR interacted with phospholipids between its active groups and the phospholipid polar groups to obtain the amphiphilic CUR-SPC complex. The multiple weak molecular interactions also resulted in high drug-loading capacity of CUR-SPC complex self-assembled NPs. The drug-loading content of the FA-PEG-CUR-SPC NPs was determined as high as 24.3 ± 2.1%. The drug-loading content of the PEG-CUR-SPC NPs and CUR-SPC NPs was also determined as 24.8 ± 3.4 and 27.6 ± 4.1%. Multiple CUR-SPC complex molecules orderly arranged to formulate the core domains of the FA-PEG-CUR-SPC NPs. The extra CUR-SPC complex molecules and DSPE-MPEG acted as surfactants transferred to the surface of the PEG-CUR-SPC NPs to sterically stabilize the nanoscaled system due to the surface energy reduction **(**Liu et al., 2007).

### *In vitro* drug release

**Figure 4.**
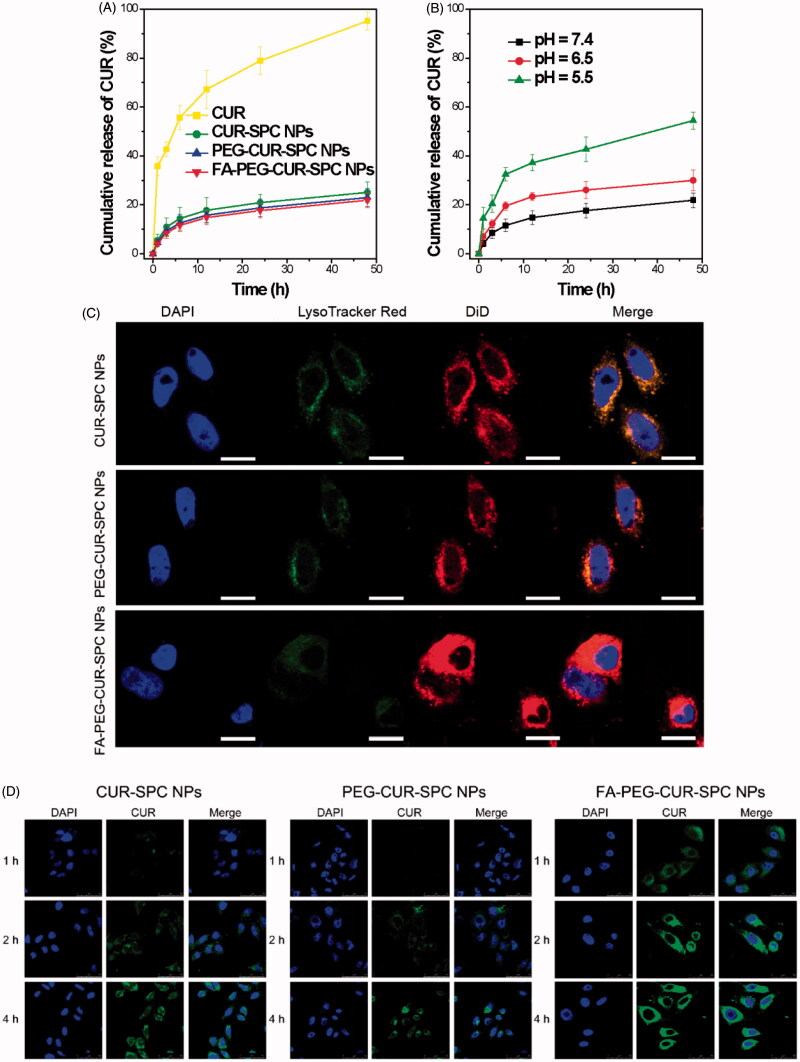
(A) *In vitro* drug release of the free CUR, CUR-SPC NPs, PEG-CUR-SPC NPs and FA-PEG-CUR-SPC NPs in PBS. Data represents mean ± SD. (*n* = 3). (B) *In vitro* pH-dependent drug release of the FA-PEG-CUR-SPC NPs in PBS. Data represents mean ± SD. (*n* = 3). (C) Lysosomal colocalization of the DiD-loaded CUR-SPC NPs, DiD-loaded PEG-CUR-SPC NPs and DiD-loaded FA-PEG-CUR-SPC NPs in HeLa cells after incubation for 12 h was observed by LCSM. The hydrophobic part of NPs was stained with DiD. The nuclei were stained with DAPI. The lysosomes were stained with LysoTracker red. (The scale bars were 25 μm). (D) *In vitro* cellular uptake of HeLa cells co-incubated with CUR-SPC NPs, PEG-CUR-SPC NPs and FA-PEG-CUR-SPC NPs examined by confocal laser scanning microscopy after 1, 2 and 4 h incubation, respectively.

The CUR release profile from the FA-PEG-CUR-SPC NPs was investigated by a dialysis method at pH 7.4 at 37 °C. Compared with the free CUR, a reduced burst release followed by a sustained release was observed in the CUR-SPC NPs, PEG-CUR-SPC NPs and FA-PEG-CUR-SPC NPs (Figure 4A). The result was possibly driven by diffusion-controlled mechanism: CUR molecules were detached from CUR-SPC complex distributed within the NPs first, and then the free CUR molecules diffused from the NPs. The drug-phospholipid complex technique not only acted as an effective platform of drug-loading but also served as a physical barrier to drug release.

The pH of tumor tissue is much lower than normal tissue as a result of lactic acid produced by hypoxia and acidic intracellular organelles (Danhier et al., 2010). Thus, the CUR release profile of the FA-PEG-CUR-SPC NPs was also investigated at pH 7.4, 6.5 and 5.5 at 37 °C, which, respectively, mimicked the physiological pH in blood circulation and normal tissues, the mildly acidic pH in tumor microenvironment and the acidic pH in intracellular endo/lysosomes. It was found that CUR release from the FA-PEG-CUR-SPC NPs was much lower at pH 7.4 (approximately 20%) than at pH 5.0 (nearly 50%) over a 24 h period (Figure 4B). This result was likely ascribed to the ionization of CUR as well as decreased mutual interaction between CUR and SPC (Acharya & Park, 2006). Based on the platform of FA-PEG-CUR-SPC NPs, CUR molecules were less released in the circulation. And as the external pH is decreased, there was a rapid CUR release in the acidic endosomes and lysosomes inside tumor cells (Figure 4C), thus this controlled release behavior showed great potential in biological stimulus-mediated targeted drug delivery.

### Subcellular location

To evaluate the intracellular CUR delivery of the different CUR formulations DiD as a lipophilic fluorescence probe was encapsulated within the hydrophobic part of NPs. HeLa cells were treated with DiD-loaded FA-PEG-CUR-SPC NPs, DiD-loaded PEG-CUR-SPC NPs, and DiD-loaded CUR-SPC NPs for 4 h. The intracellular delivery of different DiD-loaded CUR formulations is shown in Figure 4(C). The cells are stained with DAPI. All different DiD-loaded CUR formulations were found to be distributed inside the lysosomes, as evidenced by the yellow spots in the merged image obtained from the images of lysosomes (false-color green) and DiD-loaded NPs (false-color red). Thus, it was reasonable to believe that the CUR-SPC complex self-assembled NPs were internalized via the endocytosis pathway into the endosomes and lysosomes inside HeLa cells.

### *In vitro* cellular uptake

**Figure 5.**
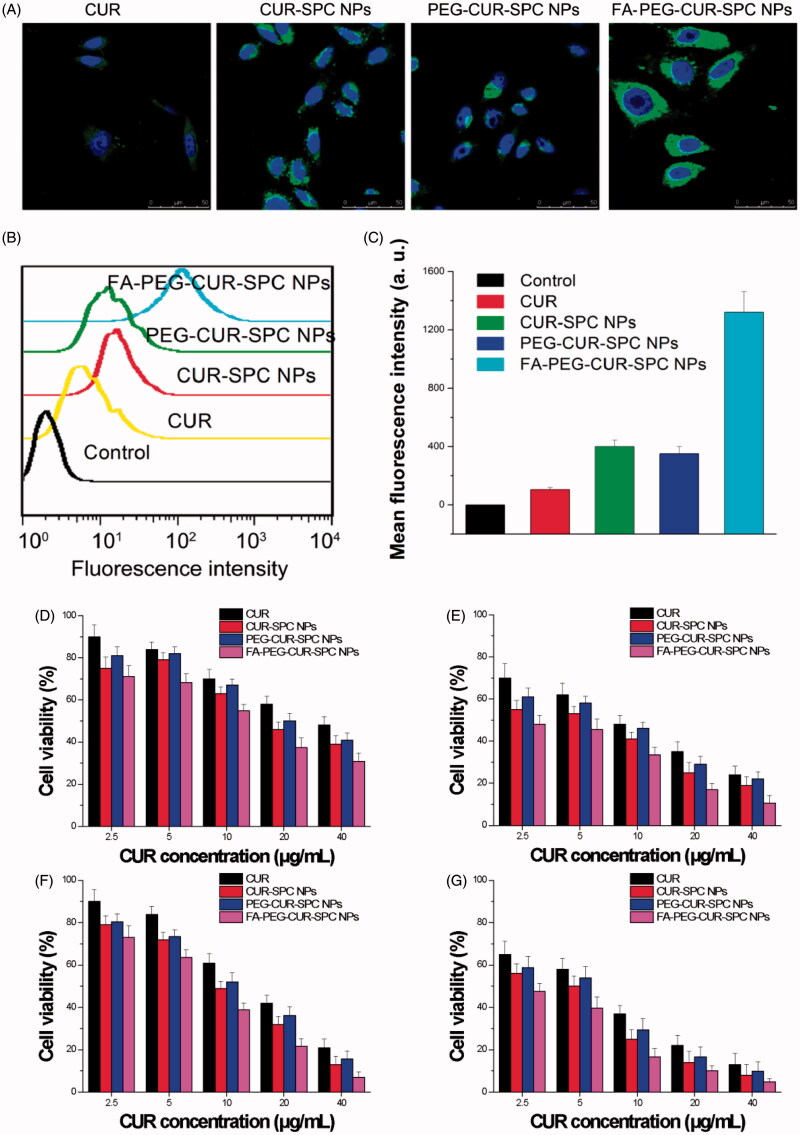
(A) *In vitro* cellular uptake of HeLa cells incubated with free CUR, CUR-SPC NPs, PEG-CUR-SPC NPs and FA-PEG-CUR-SPC NPs examined by confocal laser scanning microscopy after 4 h incubation. Blue signal, DAPI; green signal, CUR. (B) Flow cytometry histogram profiles of HeLa cells incubated with free CUR, CUR-SPC NPs, PEG-CUR-SPC NPs and FA-PEG-CUR-SPC NPs for 4 h. (C) Quantitation of mean fluorescent intensity of free CUR, CUR-SPC NPs, PEG-CUR-SPC NPs and FA-PEG-CUR-SPC NPs in HeLa cells by flow cytometry. Data were presented as mean ± SD. (*n* = 3, *p <* *0.05). (D, E) *In vitro* cell viability of HeLa cells incubated with free CUR, CUR-SPC NPs, PEG-CUR-SPC NPs and FA-PEG-CUR-SPC NPs at different concentrations (0.01, 0.05, 0.1, 0.5 and 1 mg/mL) for (D) 24 and (E) 48 h. (F, G) *In vitro* cell viability of Caco-2 cells incubated with drug-free CUR-SPC NPs and drug-free PEG-CUR-SPC NPs at different concentrations (0.01, 0.05, 0.1, 0.5 and 1 mg/mL) for (F) 24 and (G) 48 h.

CUR has intrinsic fluorescent characteristic, which also provide an available and simple way to directly track cellular uptake of CUR drug delivery system. Human cervical cancer cell lines HeLa cells with overexpressed FA receptors on the cell surfaces was chosen to address the cellular uptake and internalization behavior of the FA-PEG-CUR-SPC NPs. HeLa cells were incubated with CUR-SPC NPs, PEG-CUR-SPC NPs and FA-PEG-CUR-SPC NPs for 1, 2, and 4 h and observed by confocal laser scanning microscopy (Figure 4D). It was obviously found that the cellular uptake of different CUR formulations was time-dependent. Moreover, the greater green fluorescence intensity was clearly observed in HeLa cells after incubated with the PEG-CUR-SPC NPs and CUR-SPC NPs compared with HeLa cells incubated with the free CUR (Figure 5A). This result was well consistent with the quantitative result determined by flow cytometry (Figure 5B and C). The much higher intracellular CUR intensity of the CUR-SPC NPs and PEG-CUR-SPC NPs over the free CUR was possibly due to the enhanced uptake of CUR encapsulated within NPs because of similar structure component and high affinity between the phospholipid on the NPs’ surface and cell membranes. However, compared to the CUR-SPC NPs, the PEG-CUR-SPC NPs had a slight decrease in cellular uptake, which could be ascribed to the increase on hydrophilicity of NPs.

More importantly, compared to the free CUR, CUR-SPC NPs and PEG-CUR-SPC NPs, the FA-PEG-CUR-SPC NPs showed a significant enhancement in cellular uptake. This result confirmed the successful surface functionalization of FA on the surface of CUR-SPC complex self-assembled NPs. Therefore, both qualitative results observed by confocal laser scanning microscopy and quantitative results measured by flow cytometry proved that the internalization of the FA-PEG-CUR-SPC NPs was further increased via selective receptor-ligand mutual interactions by FA receptor-mediated endocytosis.

### *In vitro* cytotoxicity

To evaluate the tumor cell-killing efficiency of the FA-PEG-CUR-SPC NPs, HeLa cells (cervical cancer cells) and Caco-2 cells (colon cancer cells with overexpressed FA receptors on the cell surfaces) were, respectively, treated with free CUR, CUR-SPC NPs, PEG-CUR-SPC NPs and FA-PEG-CUR-SPC NPs for different incubation time periods (24 and 48 h). The cell viability was evaluated by a standard MTT assay. Compared to the free CUR, the CUR-SPC NPs and PEG-CUR-SPC NPs showed significantly enhanced cytotoxicity toward HeLa cells and Caco-2 cells (Figure 5D–G). In addition, it was found that all drug-free NPs had no significant cytotoxicity against HeLa cells and MCF-7 cells (Figure S2 in the Supporting Information), indicating that the cytotoxicity of the CUR-SPC complex self-assembled NPs was induced by the released CUR and not by the toxic effect of carrier materials. Combined with the cellular uptake results (see Figure 5A–C), these results gave us strong evidence that the greater cytotoxic effect of CUR-SPC NPs and PEG-CUR-SPC NPs was due to the enhanced cellular uptake of CUR when encapsulated within CUR-SPC complex self-assembled NPs.

Most importantly, the FA-PEG-CUR-SPC NPs displayed significantly greater cytotoxicity compared with all other CUR formulations (free CUR, the CUR-SPC NPs and even PEG-CUR-SPC NPs). For HeLa cells, the half-maximal inhibitory concentration (IC_50_) value of free CUR, the CUR-SPC NPs, and even PEG-CUR-SPC NPs and FA-PEG-CUR-SPC NPs against was determined as 32.3, 20.1, 23.9 and 11.9 μg/mL, respectively, for 24 h of incubation (8.4, 4.2, 6.3 and 2.9 μg/mL, respectively, for 48 h of incubation). For Caco-2 cells, the IC_50_ value of free CUR, the CUR-SPC NPs, and even PEG-CUR-SPC NPs, and FA-PEG-CUR-SPC NPs against was determined as 14.3, 8.9, 10.2 and 6.4 μg/mL, respectively, for 24 h of incubation (5.4, 3.6, 4.3 and 2.6 μg/mL, respectively, for 48 h of incubation). This enhancement of tumor cell-killing efficiency was caused by the enhanced and specific cellular internalization of CUR-loaded NPs by FA receptor-mediated endocytosis (see Figures 4D and 5A–C). The CUR-SPC complex self-assembled NPs with high drug-loading capacity could easily and selectively enter tumor cells, in which CUR was released to reach effective drug concentration and kill the tumor cells effectively.

### *In vivo* pharmacokinetic study

**Figure 6.**
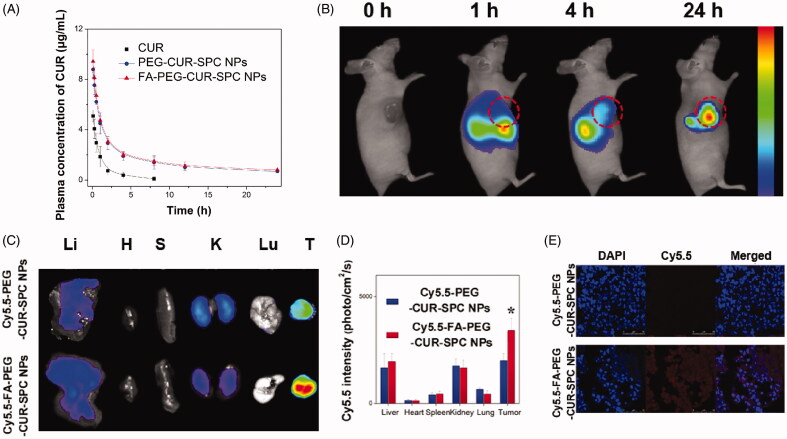
(A) *In vivo* pharmacokinetics profiles after intravenous injection of the free CUR, PEG-CUR-SPC NPs, FA-PEG-CUR-SPC NPs in rats. Data are presented as mean ± SD. (*n* = 3). (B) *In vivo* fluorescence imaging of HeLa tumor-bearing nude mice treated with Cy5.5-FA-PEG-CUR-SPC NPs by intravenous injection. Cy5.5 as a hydrophobic dye was encapsulated within the FA-PEG-CUR-SPC NPs. (C) *Ex vivo* fluorescence imaging and (D) quantitative analysis of normal organs and tumor excised from HeLa tumor-bearing nude mice treated with Cy5.5-PEG-CUR-SPC NPs and Cy5.5-FA-PEG-CUR-SPC NPs at the same concentration of Cy5.5. Li: liver; H: heart; S: spleen; K: kidney; Lu: lung; T: tumor. Data are presented as mean ± SD. (*n* = 3). (E) Frozen sections of tumors excised from mice at 24 h post intravenous injection of Cy5.5-PEG-CUR-SPC NPs and Cy5.5-FA-PEG-CUR-SPC NPs. Blue signal, DAPI. Red signal, Cy5.5.

Because of poor physiological stability of CUR-SPC NPs (see Figure S1 in the Supporting Information), we chose PEG-CUR-SPC NPs and FA-PEG-CUR-SPC NPs for *in vivo* evaluations. The mean plasma concentration versus time profiles of CUR after intravenous injection of free CUR, PEG-CUR-SPC NPs and FA-PEG-CUR-SPC NPs are shown in Figure 6(A). It was observed that the *in vivo* circulation time of FA-PEG-CUR-SPC NPs and FA-PEG-CUR-SPC NPs was obviously extended compared with the free CUR. The results indicated the reduced clearance and prolonged systemic retention effect of the FA-PEG-CUR-SPC NPs and PEG-CUR-SPC NPs in blood circulation, which might facilitate the preferential accumulation in the tumor tissues through the EPR effect **(**Li et al., 2014).

### *In vivo* tumor-targeting imaging

Cy5.5, a near-infrared lipophilic and hydrophobic fluorescent dye, was used to be encapsulated with in the FA-PEG-CUR-SPC NPs to formulate the Cy5.5 labeled FA-PEG-CUR-SPC NPs (designed as Cy5.5-FA-PEG-CUR-SPC NPs). The HeLa tumor-bearing mice were intravenously injected with the Cy5.5-FA-PEG-CUR-SPC NPs for *in vivo* fluorescence imaging. The real-time biodistribution and tumor accumulation at 1, 4 and 24 h is shown in Figure 6(B). After 0.5 h post-injection, a strong fluorescence signal was observed in liver. As time elapsed, the fluorescence signal was gradually accumulated in the tumor tissues after 4 h post-injection. The fluorescence signal at the tumor sites continued to increase after 24 h post-injection. The result indicated the preferential tumor accumulation of the Cy5.5-FA-PEG-CUR-SPC NPs. In addition, the *ex vivo* images of normal organs and tumor tissues excised form HeLa tumor-bearing mice is shown in Figure 6(C). The Cy5.5-FA-PEG-CUR-SPC NPs group exhibited the significantly superior tumor accumulation compared with the Cy5.5-PEG-CUR-SPC NPs group (Figure 6C and D). This result was well consistent with the confocal laser scanning microscopy images of frozen section of tumor tissue excised from mice (Figure 6E) and the direct assay of tissue distribution of CUR drug (Figure S3). Therefore, these results were likely ascribed to both the EPR effect and FA receptor-co-mediated passive-plus-active tumor targeting mechanism. All findings suggested that these nanoscaled CUR delivery systems showed excellent tumor-specific targeting efficiency.

## Conclusions

In our study, a simple and easy but successful method was developed to construct novel amphiphilic CUR-SPC complex self-assembled FA targeted PEGylated NPs. The FA-PEG-CUR-SPC NPs had improved characteristics including smaller particle size, relatively high-surface charge, high drug-loading capacity and great physiological stability. More importantly, the FA-PEG-CUR-SPC NPs displayed excellent *in vitro* cellular uptake/tumor cell killing efficiency and *in vivo* prolonged blood circulation time/enhanced tumor accumulation compared with PEG-CUR-SPC NPs, CUR-SPC NPs and free CUR. In view of all the advantages mentioned above, this anticancer drug-phospholipid complex self-assembled targeted nanoscaled systems could, therefore, serve as a promising nanoplatform for drug delivery of CUR. Future work will be needed for *in vivo* anticancer activity of the FA-PEG-CUR-SPC NPs for possible clinical trials.

## Notes

**Supplementary material available online** Supplementary Table S1. Supplementary Figures S1–S3.
